# Genome-wide association studies of Alzheimer’s disease and related disorders stratified by sex, onset age, and Apolipoprotein E genotype reveal novel risk loci in African Americans

**DOI:** 10.1186/s13195-025-01782-y

**Published:** 2025-07-24

**Authors:** Richard Sherva, Congcong Zhu, Rui Zhang, Jesse Mez, Richard Hauger, Victoria C. Merritt, Matthew Panizzon, J. Michael Gaziano, Vidriana Catanzaro, Gerard D. Schellenberg, Margaret Pericak-Vance, Jonathan L. Haines, Li-San Wang, Richard Mayeux, VA Million Veteran Program, J. Michael Gaziano, Sumitra Muralidhar, Jennifer Moser, Jennifer E. Deen, Philip S. Tsao, Sumitra Muralidhar, Elizabeth Hauser, Amy Kilbourne, Michael Matheny, Dave Oslin, Deepak Voora, Philip S. Tsao, Jessica V. Brewer, Mary T. Brophy, Kelly Cho, Lori Churby, Scott L. DuVall, Saiju Pyarajan, Robert Ringer, Luis E. Selva, Shahpoor Shayan, Brady Stephens, Stacey B. Whitbourne, Themistocles L. Assimes, Adriana Hung, Henry Kranzler, Lindsay A. Farrer, Mark W. Logue

**Affiliations:** 1 National Center for PTSD, VA Boston Healthcare System, Boston, MA USA; 2 Biomedical Genetics, Boston University Chobanian & Avedisian School of Medicine, Boston, MA USA; 3 Department of Neurology, Boston University Chobanian & Avedisian School of Medicine, Boston, MA USA; 4 Alzheimer’s Disease Research Center, Boston University Chobanian & Avedisian School of Medicine, Boston, MA USA; 5 Center of Excellence for Stress and Mental Health (CESAMH), VA San Diego Healthcare System, San Diego, CA USA; 6 Center for Behavioral Genetics of Aging, University of California San Diego, La Jolla, CA USA; 7 Department of Psychiatry, School of Medicine, University of California San Diego, La Jolla, CA USA; 8 Million Veteran Program (MVP) Coordinating Center, VA Boston Healthcare System, Boston, MA USA; 9 Division of Aging, Brigham & Women’s Hospital, Harvard Medical School, Boston, MA USA; 10 Department of Pathology and Laboratory Medicine Perelman School of Medicine at the University of Pennsylvania, Philadelphia, PA USA; 11 The Dr John T Macdonald Foundation Department of Human Genetics, Miller School of Medicine, University of Miami, Miami, FL USA; 12 Department of Population and Quantitative Health Sciences, School of Medicine, Case Western Reserve University, Cleveland, OH USA; 13 Columbia University Department of Neurology, New York, NY USA; 14 Bioinformatics Program, Boston University, Boston, MA USA; 15 Department of Ophthalmology, Boston University Chobanian & Avedisian School of Medicine, Boston, MA USA; 16 Department of Epidemiology, Boston University School of Public Health, Boston, MA USA; 17 Department of Biostatistics, Boston University School of Public Health, Boston, MA USA; 18 Department of Psychiatry, Boston University Chobanian & Avedisian School of Medicine, Boston, MA USA

**Keywords:** Alzheimer’s disease, Genome-wide association study, African Ancestry, RNA-sequencing, Stratified

## Abstract

**Background:**

Alzheimer’s disease (AD) risk variants have been identified in European ancestry cohorts that have stronger effects at certain ages, in individuals with a specific sex, or in those with specific isoforms of *APOE*, the strongest AD risk locus. However, sample sizes in African ancestry (AA) cohorts have been underpowered to perform stratified analyses.

**Methods:**

We generated genome-wide association study datasets stratified by sex, age at onset (< 75 vs ≥ 75), and *APOE*-ε4 carrier status in AA cohorts from MVP and the Alzheimer’s Disease Genetics Consortium (ADGC). Outcomes in MVP were AD and related dementias (ADRD; n = 4073 cases and 19,648 controls) and proxy dementia (i.e., reported dementia in a parent, *n* = 6216 cases and 21,566 controls) while ADGC analyses examined AD (*n* = 2425 cases and 5069 controls). The proxy dementia GWASs were included in the sex-stratified meta-analysis corresponding to the sex of the affected parent. The top genes were tested for differential expression in AA brain tissue.

**Results:**

In addition to the *APOE* region, genome-wide significant associations were observed in an intergenic region near the *EPHA5* gene (rs141838133, *p* = 2.19 × 10^–8^) in individuals with onset < 75 years, in *GRIN3B* near the known AD risk gene *ABCA7* (rs115882880, *p* = 3.83 × 10^–8^) in females, and near *TSPEAR* (rs139130053, *p* = 4.27 × 10^–8^) in *APOE*-ε4 non-carriers. EPHA5 regulates glucose homeostasis, and ephrin receptors modify the strength of existing synapses in the brain and in pancreatic islets. It is unclear whether *GRIN3B* represents a locus distinct from *ABCA7*. Rs115882880 was a significant eQTL for *GRIN3B* but not *ABCA7* in AA brain samples. *TSPEAR* regulates Notch signaling but has not been linked to neuronal function.

**Conclusions:**

Age, sex, and *APOE*-stratified analyses of dementia in AA participants from two cohorts revealed potential new associations. Stratified analyses may yield critical information about the genetic heterogeneity underlying dementia risk and lead to advances in precision medicine.

**Supplementary Information:**

The online version contains supplementary material available at 10.1186/s13195-025-01782-y.

## Introduction

The higher prevalence of Alzheimer’s disease (AD) in African ancestry (AA) cohorts compared to populations of European (EUR) ancestry [1, 2] is likely due to a combination of societal inequalities, factors related to comorbidities that affect AD risk, and genetics. However, despite ongoing initiatives to increase representation of AAs in genetic studies of AD and related dementias (ADRD), the sample sizes of AA and other non-EUR groups available for genetic studies of ADRD are relatively small and underpowered for genome-wide studies [3]. As a result, the number of established AD risk loci in AAs is currently a fraction of those identified in EURs [4–6]. This disparity limits our ability to understand the genetic basis of ADRD in AAs and develop genetics-guided treatment strategies that may be unique to AAs. Moreover, since the causal variants in genes associated with AD across ancestry groups often differ, knowledge gained from the collective group of disease risk variants may increase understanding about causal mechanisms that could be exploited for designing therapeutic approaches. Two examples include the genes sortilin related receptor 1 (*SORL1* [7, 8]) and ATP binding cassette subfamily A member 7 (*ABCA7* [5]), which have distinct risk alleles within ancestry groups.

Despite these challenges, substantial progress has been made in understanding the genetic architecture of AD/ADRD risk in AAs [9]. The advent of the U.S. Department of Veterans Affairs (VA) Million Veteran Program (MVP) substantially increased the sample size available for AD/ADRD genetic studies in AAs. A genome-wide association study (GWAS) of ADRD that included AA participants in MVP and the Alzheimer’s Disease Genetics Consortium (ADGC) [10] identified genome-wide significant (GWS) associations with variants in genes previously linked to AD risk (apolipoprotein E (*APOE*)*, ABCA7,* triggering receptor expressed on myeloid cells 2 (*TREM2*)*,* CD2-associated protein (*CD2AP*)) and one novel gene roundabout guidance receptor 1 (*ROBO1*) [5].

Sex, onset age, and *APOE* genotype affect the overall and/or genetic risk of AD. Many studies have explored sex differences in AD risk and presentation [11] (recently reviewed in [12]). Women have an increased risk of AD, an effect that may be partially influenced by a sex specific effect of *APOE* genotype [13]. There are also well documented differences in disease onset, progression, presentation, and pathology based on *APOE* genotype [14]. Highly penetrant rare variants in amyloid precursor protein (*APP*)*,* and presenilin 1 and 2 (*PSEN1, PSEN2*)*,* and *SORL1* are associated with early-onset AD (onset age < 60 years) and often in an autosomal dominant pattern, but other variants in these genes are associated with the common form of late-onset AD (onset age > 60 years). Most of the established AD risk genes are associated with late-onset AD. In studies of EUR ancestry populations, evidence of association with some loci for AD and AD-related traits is greatly influenced by sex [15, 16], onset age [17], disease stage [18], and *APOE* genotype [19–21]. A GWAS conducted in the ADGC AA cohorts identified GWS associations that were apparent in individuals with or without an *APOE*-ε4 allele [10]. Here, we report findings from a GWAS including data from the MVP and ADGC AA cohorts among individuals stratified by sex, onset age (< 75 years vs. ≥ 75 years), and *APOE*-ε4 status. These analyses include a small increase in the number of MVP ADRD cases from the unstratified analyses previously published (*N* = 61) and utilize a larger reference panel for SNP imputation (TopMed).

## Methods

### MVP subjects and diagnostic classification procedures

The MVP cohort and methods for defining ADRD are described in detail elsewhere [22–24]. Briefly, MVP participants are former US military service members who obtain their healthcare in the VA healthcare system who consented to allow researchers to link genetic data obtained from their biospecimens to their VA electronic medical records (EMR) for the purpose of identifying genetic risk factors for a variety of diseases and other traits. MVP participants also complete surveys related to military experiences, lifestyle factors, and health histories of themselves and their parents, including questions about AD or dementia [23].

This study included individuals who were identified genetically as AA using the Harmonized Ancestry Race Ethnicity (HARE) method [25]. ADRD cases for this study included persons with first reported ICD code ages 60 and older who had two or more ICD codes for AD or other type of dementia (i.e., frontal–temporal dementia, vascular dementia, or Lewy body dementia). Supplementary Fig. 1 shows all ICD codes and criteria used for ADRD diagnosis. After exclusions for relatedness and other considerations (described in detail below), 4,073 participants met these criteria for ADRD and were included in the genetic analyses. Because the number of dementia-free participants in MVP greatly exceeds the number of cases, potential controls were divided into two groups, one for the ADRD case/control analysis and a second for inclusion in the “proxy” ADRD GWAS described below. Different age cutoffs for cases and controls were used to maximize power and minimize misclassification. MVP AAs have a substantially larger proportion of cases with onset age between 60 and 65 compared to MVP EURs and we wanted to retain these individuals while minimizing the number of controls who were later diagnosed with ADRD. A total of 19,648 individuals, equaling approximately 4.5 times the number of ADRD cases, who were ages 65 and older without a recorded history of dementia, MCI, or history of AD medication prescription, and who did not report a parental history of dementia, were randomly selected as controls for the ADRD GWAS. Proxy AD/ADRD cases self-reported a history of dementia in their fathers or mothers and were included in the paternal or maternal proxy ADRD GWAS, respectively. Individuals with two affected parents were included in both the maternal and paternal proxy ADRD analyses. Participants aged 45 and older who didn’t report either parent as having dementia were included as controls in the proxy analysis. Participants included in the ADRD GWAS were excluded from the sample for the proxy dementia/ADRD GWAS so that ADRD and proxy analyses are independent. After applying these criteria, 4,385 maternal proxy cases, 2,256 paternal proxy cases, and a common control proxy cohort of 45,970 subjects remained for analysis.

### ADGC subjects and diagnostic classification procedures

Ascertainment and diagnostic procedures applied to participants in the ADGC cohorts have been described previously [10]. Briefly, multiple cohorts with different methodologies comprise the full sample. It contains both family-based and case–control cohorts with a substantial number of autopsy-confirmed cases. All controls were screened for AD. The study included subjects from the Adult Changes in Thought Study, the National Institute on Aging Alzheimer ‘s Disease Centers, the University of Miami/Vanderbilt University, the Mount Sinai School of Medicine Brain Bank, the Washington Heights Inwood Columbia Aging Project, The African American Alzheimer's Disease Genetics Study, the MIRAGE Study, NIA- LOAD/NCRAD, the Mayo Clinic, the Rush University Alzheimer’s disease Center, the Chicago Health and Aging Project, the Indianapolis Ibadan Dementia Study, the Genetic and Environmental Risk Factors for Alzheimer’s Disease Among African Americans Study, the University of Pittsburgh, and Washington University. All subjects were recruited under protocols approved by the appropriate Institutional Review Boards. These combined cohorts included 2,425 AD cases and 5,069 controls in total, representing 751 male cases, 1,674 female cases, 1,276 APOE-ε4 positive cases, 851 APOE-ε4 negative cases, 1,011 cases with onset < 75 years, and 1,414 cases with onset ≥ 75 years.

### Genotype data generation, quality control, and SNP imputation procedures and population structure analysis

Genotype data processing and cleaning was performed by the MVP Bioinformatics core using the MVP 1.0 custom Axiom array [26]. Quality control (QC) included checks for sex concordance, advanced genotyping batch correction, and assessment for relatedness. Genotyping in the ADGC cohorts was performed using various microarrays and these data were processed using methods described elsewhere [10]. After extensive QC, SNP genotypes were imputed in both MVP and ADGC datasets using the TopMed r2 imputation panel, SHAPEIT4 version 4.1.3 for phasing, and MINIMAC version 4. Related individuals in the MVP dataset defined as a kinship coefficient of 0.09375 or higher were removed from analysis. When both members of a pair were cases, we selected a subject with AD-specific ICD codes or, in the absence of ICD codes, one individual was randomly selected. In MVP, principal components (PC) of ancestry were computed with FlashPCA version 2 [27], using only AA individuals and a linkage-disequilibrium pruned set of 170,207 SNPs that excluded the major histocompatibility complex region of chromosome 6. In the ADGC cohorts, to identify outlier samples within each dataset with ancestry group, we performed a PC analysis using ‘smartpca’ in EIGENSOFT version 5.0 for the subset of ~ 20,000 LD-pruned SNPs used for relatedness checks on genotypes from all samples within each individual dataset and from the 1kG Phase 3 reference panels. Individuals not clustering with their reported ancestry groups (or between reported ancestry groups for admixed subjects) were excluded from analysis when including 1KG groups. The PCs used for analysis were generated in the remaining AA individuals. *APOE* genotypes were determined using the “best guess” imputed genotypes (80% confidence threshold) for the rs7412 and rs429358 SNPs. SNPs with minor allele frequency (MAF) > 1% and imputation quality (r^2^) > 0.4 were included in each analysis.

### Statistical and bioinformatic analysis methods

In both the ADGC and the MVP cohorts, GWAS were conducted within six strata of the data: males and females, those with ADRD/AD onset age < 75 years vs. ≥ 75 years, and *APOE* ε4 allele carriers and non-carriers. The onset age cutoff was chosen for two reasons: first, that is the cutoff that was previously being used for onset based genetic analyses and second, that cutoff approximately split the case sample into two evenly sized bins. All MVP controls were ≥ 65 years of age at last visit and a common set of controls were included for each stratum (i.e. controls were not age selected for the onset stratified analyses). ADGC cohorts with fewer than 10 cases within each analysis stratum were excluded from that analysis. Results from the remaining 14 ADGC cohorts were combined by meta-analysis using METAL version 2020–05-05 [28] and these results were subsequently combined with those from MVP. Individuals with the *APOE* ε2/ε4 genotype were excluded from analyses stratified by ε4 carrier status. Association of each SNP with AD in the ADGC cohorts and ADRD and proxy dementia in MVP was tested using logistic regression models including terms for sex (excluding the sex-stratified analysis) and the first ten ancestry PCs implemented in PLINK 2.0. Firth logistic regression was applied when the standard regression model failed to converge. GWAS was performed separately for the MVP maternal and paternal proxy dementia outcomes using a common set of controls, and the results from each GWAS were combined via meta-analysis with the respective sex-stratified AD and ADRD GWAS results using betas weighted by the inverse of their standard errors and METAL. The result of the ADGC meta-analysis was then meta-analyzed with the corresponding MVP results. The betas and standard errors of the proxy GWASs were both multiplied by two to account for the fact that they only share half their genes with the affected parent [29]. Functional annotation, expression quantitative trait loci (eQTL) analysis, gene-based tests, gene set enrichment, and pathway analyses were performed using the FUMA web portal [30]. FUMA uses the MAGMA [31] method for gene-based and gene set enrichment tests.

### RNA-seq expression profiling and eQTL analysis

The methods used to assess variants for potential regulatory effect have been described in detail elsewhere [32]. Briefly, the potential of variants as eQTLs was assessed in pre-frontal cortex tissue from a cohort of 177 AA neuropathologically determined AD case and control donors (125 AD cases and 82 neuropathologically determined controls) assembled from 13 ADRC brain banks. Gene expression was assessed via RNA-seq. Genome-wide genotypes were assessed using the Illumina Global Diversity Array (~ 1.8 million variants) and imputed using 1000 Genomes phase 3v5 reference data [33]. We tested association between the significant variants with MAF > 5% from stratified analyses with all expressed genes within 200 kb. A regression model assessed association between the rLog expression values with the additively (0 to 2) coded variant, with covariates for RNA integrity number (RIN), age at death, sex, batch and estimated cell proportions. A false discovery rate (FDR) corrected *p*-value was computed to adjust for the number of genes examined with each variant.

## Results

**Fig. 1:**
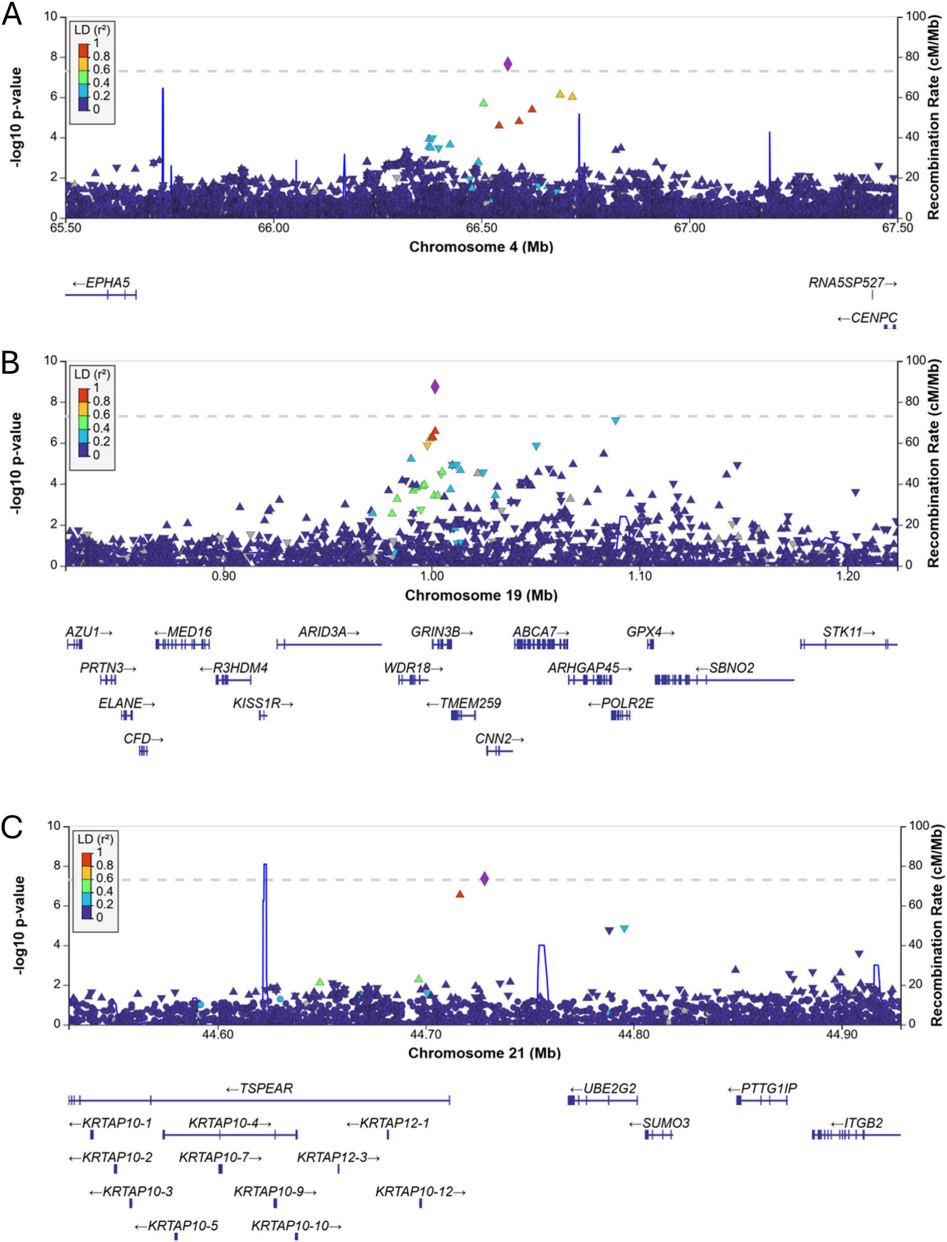
Regional Manhattan plots showing the genome wide significant associations: The *EPHA4* region on chromosome 4 identified in individuals with ADRD onset less than 75 years (**A**). The *GRIN3B*-*ABCA7* region on chromosome 19 identified in females (**B**). The *TSPEAR* region on chromosome 21 identified in individuals without an *APOE*-ε4 allele (**C**). Chromosomal position in GRCh38 is on the X axis and the Y axis shows the -log10 *p*-values for each SNP in the region. The degree of linkage disequilibrium with the peak SNP (purple diamonds) is indicated by the color of the point on the plot in the 1000 Genomes Project AFR reference panel

**Fig. 2:**
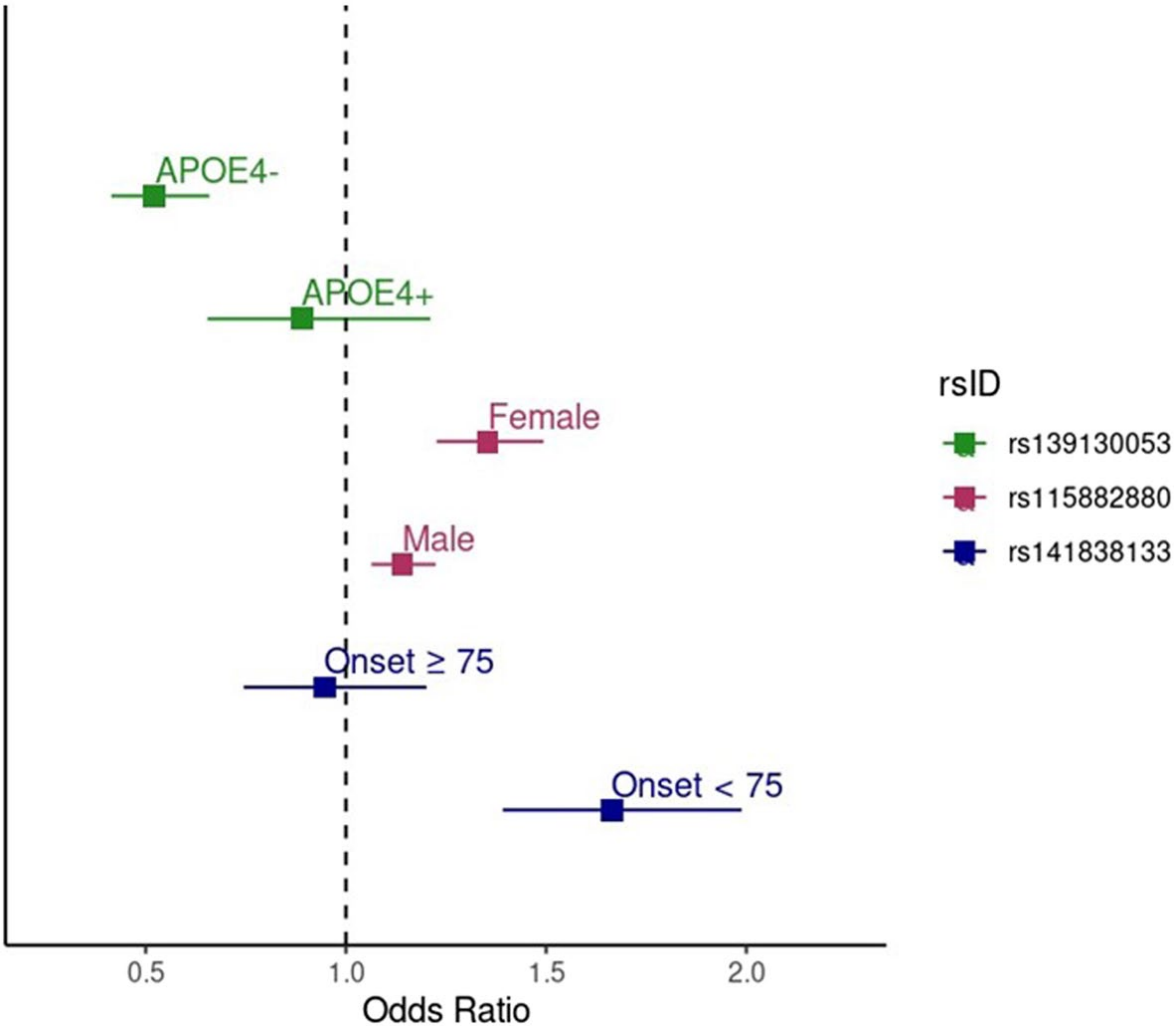
Forest plot showing the effect sizes (odds ratios) and 95% confidence intervals for the MVP/ADGC meta-analysis results for the genome-wide significant SNPs in both the stratum in which the association was identified and the opposite stratum. The green points show the effect of rs139130053 in *APOE*-ε4 negative individuals (upper green point) and *APOE*-ε4 positive individuals (lower green point). The pink points show the effect of rs115882880 in females (upper pink point) and males (lower pink point). The blue points show the effect of rs141838133 in individuals with onset age ≥ 75 years (upper blue point) and individuals with onset age < 75 years (lower blue point)

Table 1 shows the demographic characteristics of the MVP and ADGC cohorts contributing to each of the six stratified GWAS analyses. Outside the *APOE* region, we identified three genome-wide significant associations, one each in those with a censoring age < 75 years, females, and *APOE-*ε4 non-carriers, as well as suggestive associations with several additional loci, with little evidence for genomic inflation in the GWAS for each stratified group (λ < 1.05, Table 2, Supplementary Figs. 2–4, Supplementary Tables 1–6). Table 2 shows the GWS loci, including a SNP in an intergenic region near ephrin receptor A5 (*EPHA5*; rs141838133, OR = 1.66, *p* = 1.66 × 10^–8^) in individuals with onset < 75 years, in glutamate ionotropic receptor NMDA type subunit 3B (*GRIN3B*) near the known AD risk gene *ABCA7* (rs115882880, OR = 0.74, *p* = 1.8 × 10^–9^) in females, and between the genes thrombospondin type laminin G domain and EAR repeats (*TSPEAR)*;and ubiquitin-conjugating enzyme e2 g2 (*UBE2G2*) in APOE-ε4 non-carriers rs139130053, OR = 0.52, *p *= 4.39 × 10^–8^). All these SNPs showed evidence for association with the outcome in both MVP and ADGC. Figure 1 shows the regional Manhattan plots for these associations. Supplementary Tables 1–6 show results for SNPs with meta-analysis *p*-values less than 1.0 × 10^–5^ within each of the six strata, within MVP and ADGC, and the unstratified result published in [5]. Figure 2 shows the odds ratios and 95% confidence intervals (C.I.) for each SNP in both the group where it was identified and the corresponding strata. For two of the GWS associations, the 95% C.I. in the two strata do not overlap, whereas the C.I. for the ORs for rs139130053 in the *APOE-*ε4- and *APOE-*ε4 + groups overlap by 0.01. There was a nominally significant (*p* = 2.34 × 10^–4^) association in males for rs115882880, but no evidence for association (*p* > 0.05) in the opposite strata for the other two GWS associations. There was one significant female-specific gene-based test result in cyclin-dependent kinase inhibitor 2b (*CDKN2B*, *p* = 2.29 × 10^–6^).

**Table 1:** Demographic characteristics of the MVP and ADGC cohorts contributing to each analysis stratum

Cohort	Females N cases/N controls, (µ age cases/µ age controls)	Maternal Proxy N cases/N controls	Males N cases/N controls (µ age cases/µ age controls)	Paternal Proxy N cases/N controls	APOE-ε4 + N cases/N controls (µ age cases/µ age controls)	APOE-ε4- N cases/N controls (µ age cases/µ age controls)	Onset < 75 N cases/N controls (µ age onset cases/µ age controls)	Onset ≥ 75 N cases/N controls (µ age onset cases/µ age controls)
MVP	120/1,090 (67.7/70.2))	4,385/45,970	3,953/18,558 (75.5/72.0)	2,256/45,970	1,555/5,693 (74.9/72.7)	1,950/11,477 (75.3/73.2)	2,430/13,326 (63.9/73.0)	1,643/6,322 (82.5/73.0)
ADGC	1,674/3,678 (77.4/76.2)	NA	751/1,391 (76.2/76.3)	NA	1,276/1,528 (75.0/75.3)	851/3,207 (79.3/76.5)	1,011/5,069 (68.7/76.2)	1,414/5,069 (83.0/76.3)

Parental proxy cases were only available in the MVP cohort

*NA* Not available

**Table 2:** Association results in the MVP/ADGC meta-analysis for the three genome-wide significant SNPs shown in each stratum

				OR [95% C.I.] (Meta *P*-value)					
SNP	A1	A1 Freq	Gene	APOE-ε4 +	APOE-ε4 -	Female	Male	Onset < 75	Onset ≥ 75
rs141838133	T	0.02	*EPHA5*	1.15 [0.89–1.48] (2.87E-01)	1.44 [1.17–1.77] (4.61E-04)	1.10 [0.89–1.39] (3.48E-01)	1.38 [1.19–1.60] (3.19E-05)	1.66 [1.39–1.99] (**2.24E-08**)	0.95 [0.74–1.20] (6.47E-01)
rs115882880	A	0.10	*GRIN3B*	1.25 [1.12–1.39] (6.99E-05)	1.18 [1.07–1.30] (9.07E-04)	1.35 [1.23–1.49] (**1.81E-09**)	1.14 [1.06–1.22] (2.34E-04)	1.19 [1.09–1.30] (7.78E-05)	1.22 [1.10–1.35] (1.34E-04)
rs139130053	A	0.01	*TSPEAR*	0.89 [0.65–1.21] (4.60E-01)	0.52 [0.41–0.66] **(4.39E-08**)	0.89 [0.68–1.16] (3.90E-01)	0.75 [0.63–0.89] (1.31E-03)	0.74 [0.60–0.92] (6.32E-03)	0.70 [0.53–0.91] (7.00E-03)

Meta *P*-value = *P*-value from the meta-analysis of MVP ADRD, ADGC AD, and MVP maternal and paternal proxy for the female and male strata

*A1* Effect allele, *A1 Freq* Allele frequency of A1, *OR* Odds ratio for the effect of A1

Genome-wide significant *P*-values are in bold

### Functional annotation and gene set enrichment analysis

**Fig.3:**
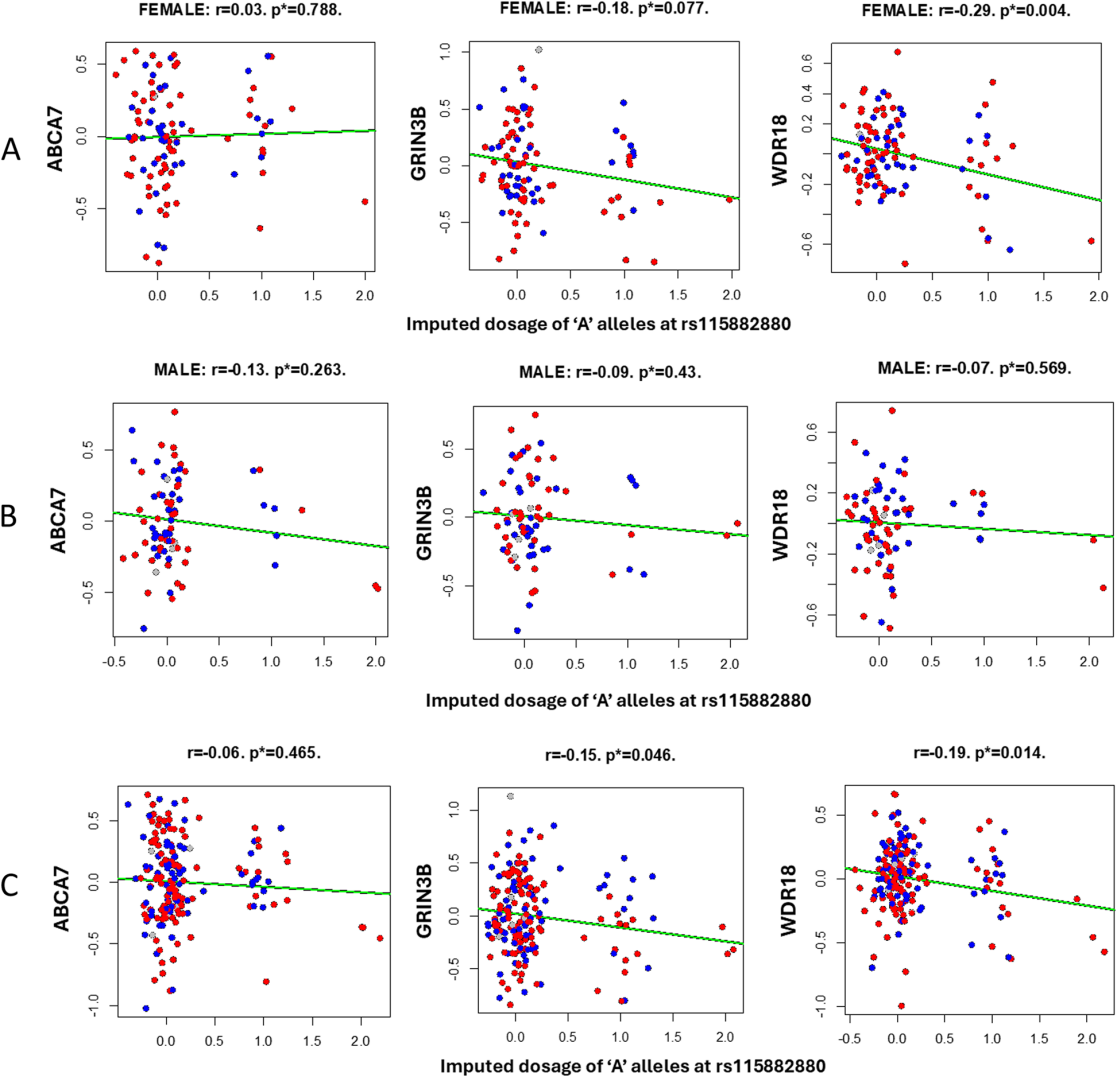
Expression levels (Y axis) of ABCA7 (first column), GRIN3B (second column), and WDR18 (third column) by imputed rs115882880 dosage (X axis) in African American brain tissue in females (**A**), males (**B**), and unstratified (**C**). The red points represent AD cases, and the blue dots represent cognitively normal controls

Several gene ontology terms and canonical pathways had significantly enriched associations, including triglyceride rich lipoprotein particle clearance (p_adj_ = 0.045) in onset < 75, the alpha defensins pathway in females (p_adj_ = 0.009), and the KEGG insulin signaling pathway in *APOE*-ε4 positives (p_adj_ = 0.025). Previously identified GO terms and pathways, primarily driven by genes in the *APOE* region on chromosome 19, were also significant in several strata (results not shown). None of the GWS SNPs showed evidence for being eQTLs in the Genotype Tissue Expression database, which is primarily comprised of individuals of EUR ancestry, or two recently published databases [34] of AA samples [35] (*N* = 1,032 and *N* = 805). Two of the SNPs (rs141838133 and rs139130053) did not appear in the AA brain expression databases, likely due to their low MAFs. The RNA-seq data generated internally showed a significant eQTL effect for rs115882880 on expression of both *GRIN3B* (r = −0.15, *p* = 0.046, Fig. 3) and WD Repeat Domain 18 (*WDR18* r = −0.12, *p* = 0.017), but not *ABCA7* (*p* = 0.46). Figure 3 shows expression levels of *GRIN3B* by rs115882880 genotype in the full sample. ‘A’ alleles were associated with lower *GRIN3B* expression. The stratified eQTL analyses showed that the eQTL effect of rs115882880 on *GRIN3B* was not significant in males and attenuated in females (*p* = 0.077), while the eQTL effect on *WDR18* was stronger in females than in the full sample (r = 0.29, *p* = 0.004) (Fig. 3). Although the peak SNP between *TSPEAR* and *UBE2G2* is not an eQTL for either of these genes, UBE2G2 itself is nominally significantly over expressed in AD vs control brains (*p* = 0.02).

## Discussion

Here, we present the results of GWAS analyses of dementia in AAs stratified by biologically relevant factors known to affect the risk and/or trajectory of ADRD. We identified two potentially novel risk variants in previously unreported genes and one potentially novel variant in a known risk locus.

It is difficult to determine whether the SNP in *GRIN3B* represents a distinct association signal from the previously identified ones in *ABCA7*. The lead SNP in females is in moderate LD (r^2^ = 0.38) with the *ABCA7* SNP identified by Reitz et al., but low LD (r^2^ < 0.03) with the lead SNP(s) identified previously in an unstratified meta-analysis of MVP and ADGC AAs [5]. The unstratified result is also highly significant, but the magnitude of the association is stronger in females and the maternal proxy cases. Conditional analysis of the *GRIN3B* SNP adjusting for the *ABCA7* SNPs is difficult to interpret since the peak *ABCA7* SNPs are different in MVP and the ADGC females (and in the unstratified analysis), and also because the results is driven by ADGC and the MVP maternal proxy analyses, the latter of which then requires two proxy variants rather than one, which may introduce an additional level of misclassification. Despite these caveats, we tested models conditional on rs115882880. In both ADGC and MVP, the results were attenuated but still significant (ADGC *p*-value = 0.0002, MVP *p*-value = 0.01). Leaving out the maternal proxy results from the meta-analysis attenuated the result as well (OR = 1.41, *p* = 2.17 × 10^–6^).

Although the eQTL results do not provide direct evidence for or against the independence of the signal in *GRIN3B* on a genetic level, the fact that it affects the expression of *GRIN3B* and *WDR18* but not *ABCA7* suggests a biological effect of the SNP itself that is independent of *ABCA7*.

Protein coding genes near the GWS SNPs have potential biological significance to AD and dementia. *EPHA5* has two functions through which it might affect risk. Eph receptors modify the strength of existing synapses in the adult brain [36], and in pancreatic islets it affects basal and glucose-stimulated insulin secretion and improves glucose homeostasis. Other Eph receptors (*EPHA1*, *EPHA4*, *EPHB*) have been previously associated with AD risk and AD-related pathology [37–42], reviewed in [43]. *GRIN3B* is a member of a gene family encoding glutamate-regulated ion channels expressed throughout the central nervous system, especially motor neurons [44], where they can have both excitatory or inhibitory effects depending on which additional subunits comprise the channels [45]. *WDR18* has not been linked to dementia relevant outcomes but is involved with many cellular processes including cell cycle progression, signal transduction, apoptosis, and gene regulation [46, 47]. Finally, *TSPEAR* functions in the Notch signaling pathway which has been previously linked to AD pathology [48–51]. *TSPEAR* itself, however, has not been implicated in AD risk and appears to function in human tooth and hair follicle morphogenesis [52], but is expressed in the adult brains of mice [53]. Expression in human brain, however, is relatively low. Alternately, the association could be driven by the gene on the other side of the peak SNP, *UBE2G2*, which has functions related to the degradation of misfolded proteins in the endoplasmic reticulum [54], which may suggest an effect on ADRD risk through tau. *UBE2G2* is expressed in several human brain regions according to GTEx. Nothing in the literature suggests why either of these genes might influence dementia risk only in, or more strongly within, the strata in which they were observed.

*CDKN2B*, the gene with a significant gene-based test result, has been previously implicated as a potential AD risk locus. First identified through linkage studies in families of EUR ancestry [55] and also consanguineous Israeli-Arab families [56], three SNPs in the gene were later associated with AD [57], but the associations were not replicated [58]. The fact that we observed an association in female individuals of AA ancestry provides further evidence that this gene may influence AD risk. Cyclin dependent kinases have been hypothesized to affect AD through mitogenic pathways such as the p21/MAPK cascade which may lead to disturbed APP processing or hyperphosphorylated tau [59].

Several of the suggestive association signals (*p* < 5.0 × 10^–7^) have potential links to AD biology. Protein phosphatase 3, regulatory subunit b, alpha (*PPP3R1*) is involved in a Ca^2+^-responsive signaling pathway and lower expression of the gene caused perturbations in numerous AD-relevant co-expression networks [60]. Variants in the gene predicted more rapid progression of AD [61]. The association was observed in females with a variant not previously linked to AD (rs113668008, *p* = 8.52 × 10^–8^). Endoplasmic reticulum oxidoreductin 1-like, beta (*ERO1B*) is involved in pro-cellular survival pathways via regulation of protein folding and is involved in insulin production. In cell lines, it is upregulated in response to Aβ and Tau expression [62]. The association signal was observed in males (rs79144457, 3.40 × 10^–7^). Also in males, variants in the gene caseinolytic mitochondrial matrix peptidase chaperone subunit (*CLCX*) were associated with dementia (rs114713526, *p* = 4.41 × 10^–7^). This protein, along with other molecules, is involved in preserving mitochondrial health through degradation of misfolded proteins [63]. In males, association was also observed with a variant in the autism risk gene intellectual developmental disorder, autosomal dominant 26 (*AUTS2*) (rs17684339, *p* = 9.59 × 10^–8^). The association with this variant was also observed in individuals with onset age less than 75 (*p* = 3.53 × 10^–7^). Several members of the A disintegrin and metalloprotease (*ADAM*) gene family have been linked to AD through genetic association and functional experiments. We identified a SNP in ADAM metallopeptidase with thrombospondin type 1 motif 19 (*ADAMTS19*) that was associated with dementia in individuals with onset less than 75 years (rs67540991, *p* = 2.27 × 10^–7^). *ADAMTS19* has primarily been linked to cardiac phenotypes [64].

### Strengths and limitations

This work had several strengths, including being the largest sample of AA individuals with genotypes and dementia diagnoses to be analyzed within biologically relevant strata, and in MVP, a control group with no reported parental dementia. Several limitations should also be noted. First, despite assembling the largest cohort of AA individuals with dementia diagnoses and genetic data, the overall power, especially after stratification, is much lower than that of European ancestry meta-analyses. This study meta-analyzed results from three different dementia phenotypes: ADRD, AD, and parental proxy dementia. Although the statistical power obtained by combining these phenotypes was likely the reason we found novel associations, we are unable to determine whether they are AD-specific. Also, the MVP samples were phenotypes using EMR data and controls were not formally screened for dementia. Finally, two of the three GWS SNPs were relatively rare (MAFs greater than 1% but less than 3%), which increases the possibility that they are false positives. Larger AA GWAS studies may provide further evidence for these SNPs’ effect on AD risk in their respective strata.

## Conclusions

These results highlight the benefits of studying ADRD genetics in non-EUR populations, as well as within biological/disease onset-defined subsets of cases due to the heterogeneity of dementia disorders. They suggest a distinct risk locus within the ABCA7 region, as well as novel risk loci near *TSPEAR* and *EPHA5*. In summary, this work provides evidence for novel dementia risk loci that are specific to individuals who are *APOE*-ε4 non-carriers, females, and have an earlier age at onset (< 75 years).

## Supplementary Information

Supplementary Material 1: Figure S2. Manhattan and quantile-quantile (QQ) plots for the age at onset stratified GWAS. The top left shows the Manhattan plot for individuals with onset < 75 years. The top right shows the QQ plot for this analysis. The bottom left shows the Manhattan plot for individuals with onset ≥ 75 years. The bottom right shows the QQ plot for this analysis. Chromosomal position in GRCh38 is on the X axis and the Y axis shows the -log10 *p*-values for each SNP for the Manhattan plots. SNPs with *P*-values < 5.0x10-9 were excluded from both Manhattan and QQ to reduce the effect of the APOE region. Figure S3. Manhattan and quantile-quantile (QQ) plots for the sex stratified GWAS. The top left shows the Manhattan plot for females. The top right shows the QQ plot for this analysis. The bottom left shows the Manhattan plot for males. The bottom right shows the QQ plot for this analysis. Chromosomal position is on the X axis and the Y axis shows the -log10 *p*-values for each SNP for the Manhattan plots. SNPs with *P*-values < 5.0x10-9 were excluded from both Manhattan and QQ to reduce the effect of the APOE region. Figure S4. Manhattan and quantile-quantile (QQ) plots for the APOE-ε4 stratified GWAS. The top left shows the Manhattan plot for individuals without any ε4 alleles. The top right shows the QQ plot for this analysis. The bottom left shows the Manhattan plot for with one or two copies of the ε4 allele. The bottom right shows the QQ plot for this analysis. Chromosomal position is on the X axis and the Y axis shows the -log10 *p*-values for each SNP for the Manhattan plots. SNPs with *P*-values < 5.0x10-9 were excluded from both Manhattan and QQ to reduce the effect of the APOE region for the ε4+ plots.

Supplementary Material 2: Supplementary tables.

## Data Availability

GWAS summary results for the MVP cohort will be posted to dbGAP after publication. The data and code used to generate MVP results are accessible to researchers with MVP data access. Due to VA policy, MVP is currently only accessible to VA researchers with an approved and funded MVP project, either through a VA Merit Award, career development award, or NIH R01 (see https://www.research.va.gov/funding/Guidance-MVP-Data-Access-Merit-Award.pdf).

## Abbreviations

AD: Alzheimer’s disease
AA: African Ancestry
EUR: European Ancestry
SORL1: Sortilin related receptor 1
ABCA7: ATP binding cassette subfamily A member 7
ADRD: Alzheimer’s disease and related dementias
MVP: Million veteran program
GWAS: Genome wise association study
GWS: Genome-wide significant
ADGC: Alzheimer’s Disease Genetics Consortium
APOE: Apolipoprotein E
TREM2: Triggering receptor expressed on myeloid cells 2
CD2AP: CD2-associated protein
ROBO1: Roundabout guidance receptor 1
APP: Amyloid precursor protein
PSEN1: Presenilin 1
PSEN2: Presenilin 2
EMR: Electronic medical records
HARE: Harmonized ancestry race ethnicity
QC: Quality control
MAF: Minor allele frequency
eQTL: Expression quantitative trait loci
RIN: RNA integrity number
FDR: False discovery rate
EPHA5: Ephrin receptor A5
GRIN3B: Glutamate ionotropic receptor NMDA type subunit 3B
TSPEAR: Thrombospondin type laminin G domain and EAR repeats
CDKN2B: Cyclin-dependent kinase inhibitor 2B
WDR18: WD Repeat Domain 18
UBE2G2: Ubiquitin-conjugating enzyme e2 g2
PPP3R1: Protein phosphatase 3 regulatory subunit b
ERO1B: Endoplasmic reticulum oxidoreductin 1-like beta
CLCX: Caseinolytic mitochondrial matrix
AUTS2: Intellectual developmental disorder autosomal dominant
ADAM: A disintegrin and metalloprotease
ADAMTS19: ADAM metallopeptidase with thrombospondin type 1 motif 19
