# Supplementary material for: Genome-wide association studies of Alzheimer’s disease and related disorders stratified by sex, onset age, and Apolipoprotein E genotype reveal novel risk loci in African Americans
Source: Alzheimers Res Ther. 2025 Jul 24;17:171. doi: 10.1186/s13195-025-01782-y (PMC12288278; doi:10.1186/s13195-025-01782-y)
Supplement: Supplementary file 1 — Supplementary Material 1: Figure S2. Manhattan and quantile-quantile (QQ) plots for the age at onset stratified GWAS. The top left shows the Manhattan plot for individuals with onset < 75 years. The top right shows the QQ plot for this analysis. The bottom left shows the Manhattan plot for individuals with onset ≥ 75 years. The bottom right shows the QQ plot for this analysis. Chromosomal position in GRCh38 is on the X axis and the Y axis shows the -log10 p-values for each SNP for the Manhattan plots. SNPs with P-values < 5.0x10^-9^ were excluded from both Manhattan and QQ to reduce the effect of the APOE region. Figure S3. Manhattan and quantile-quantile (QQ) plots for the sex stratified GWAS. The top left shows the Manhattan plot for females. The top right shows the QQ plot for this analysis. The bottom left shows the Manhattan plot for males. The bottom right shows the QQ plot for this analysis. Chromosomal position is on the X axis and the Y axis shows the -log10 p-values for each SNP for the Manhattan plots. SNPs with P-values < 5.0x10^-9^ were excluded from both Manhattan and QQ to reduce the effect of the APOE region. Figure S4. Manhattan and quantile-quantile (QQ) plots for the APOE-ε4 stratified GWAS. The top left shows the Manhattan plot for individuals without any ε4 alleles. The top right shows the QQ plot for this analysis. The bottom left shows the Manhattan plot for with one or two copies of the ε4 allele. The bottom right shows the QQ plot for this analysis. Chromosomal position is on the X axis and the Y axis shows the -log10 p-values for each SNP for the Manhattan plots. SNPs with P-values < 5.0x10^-9^ were excluded from both Manhattan and QQ to reduce the effect of the APOE region for the ε4+ plots. [file 13195_2025_1782_MOESM1_ESM.pptx]

## Slide 1
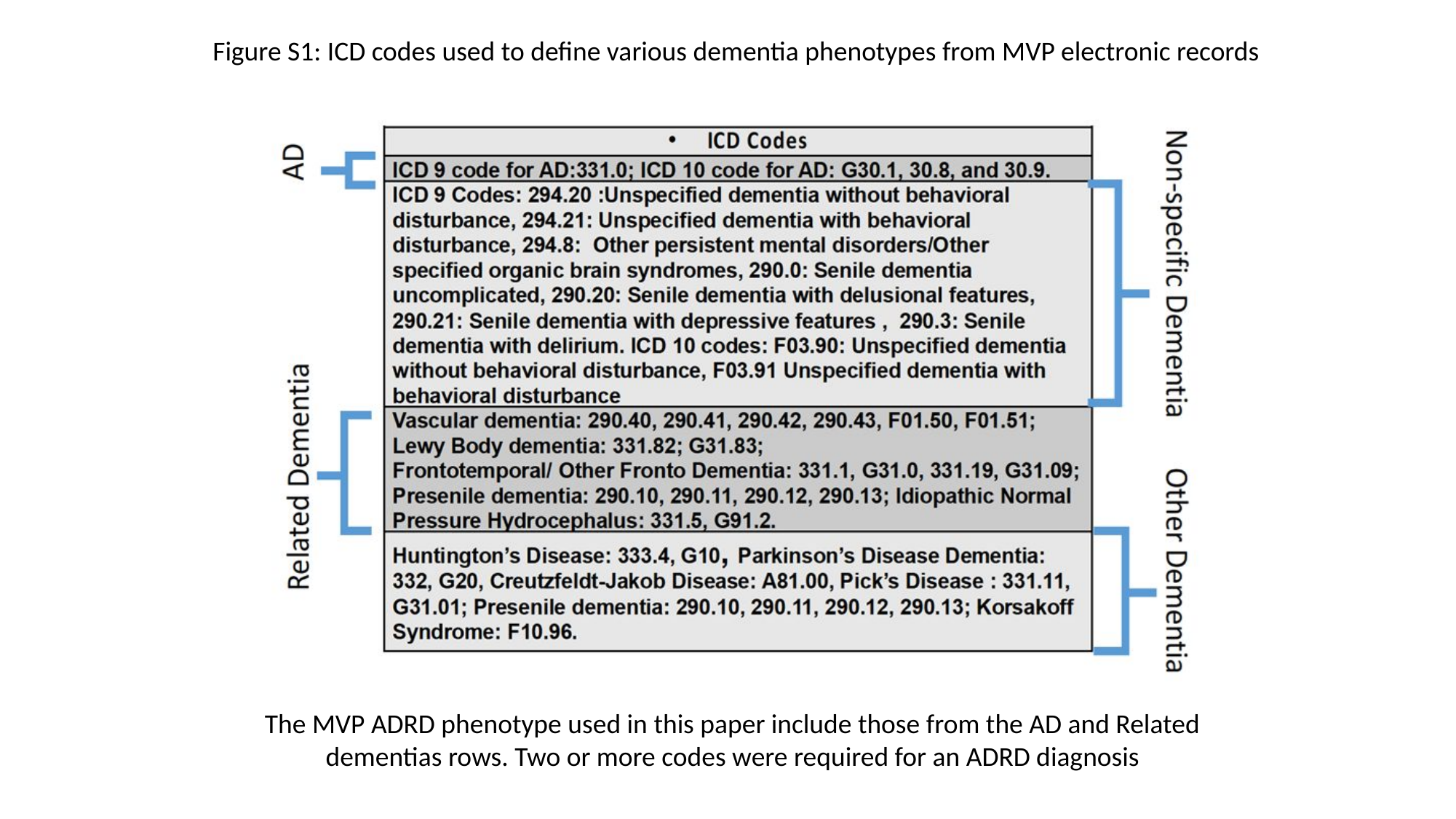

Figure S1: ICD codes used to define various dementia phenotypes from MVP electronic records
The MVP ADRD phenotype used in this paper include those from the AD and Related dementias rows. Two or more codes were required for an ADRD diagnosis

## Slide 2
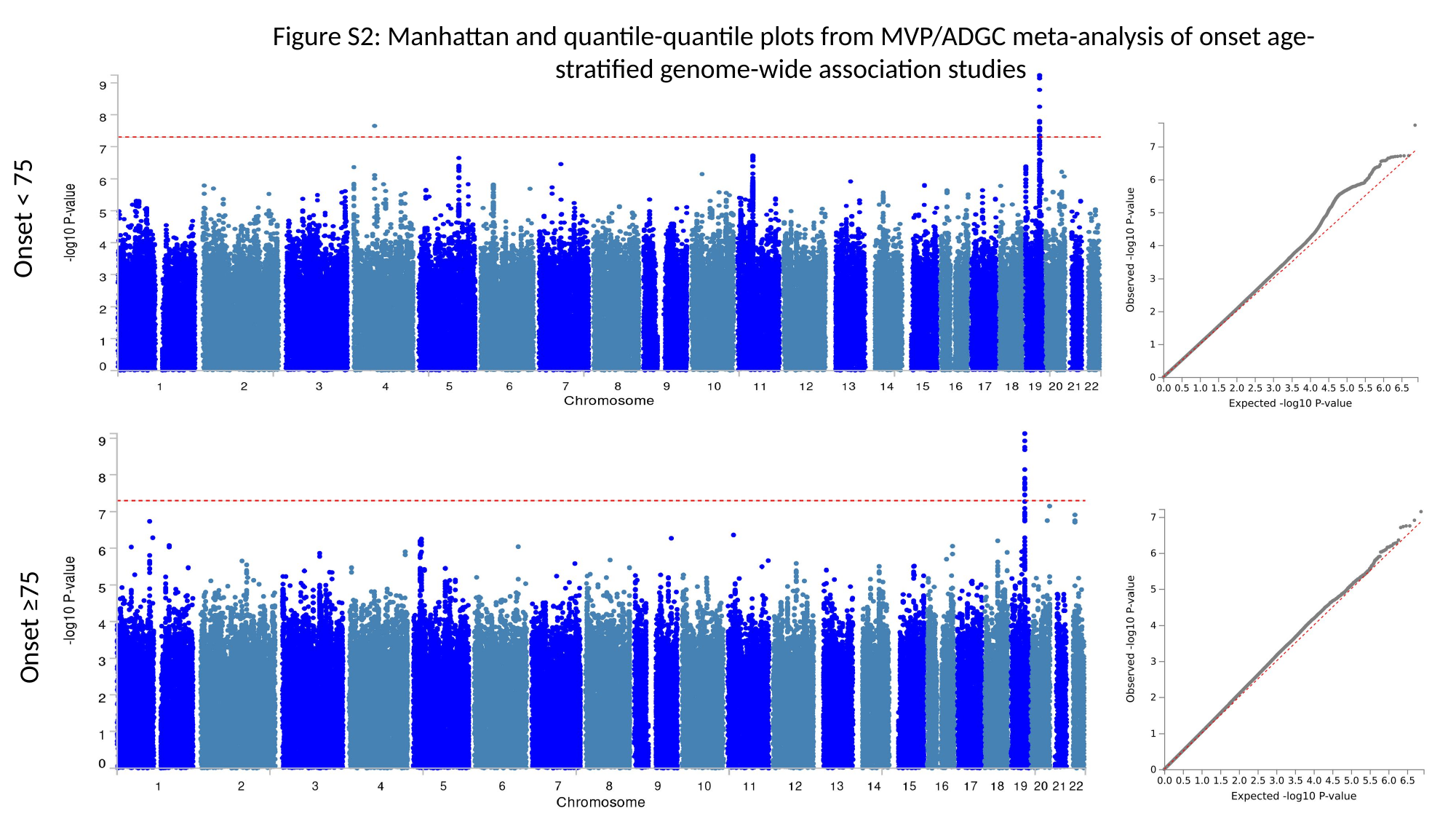

Figure S2: Manhattan and quantile-quantile plots from MVP/ADGC meta-analysis of onset age-stratified genome-wide association studies
Onset < 75
Onset ≥75

## Slide 3
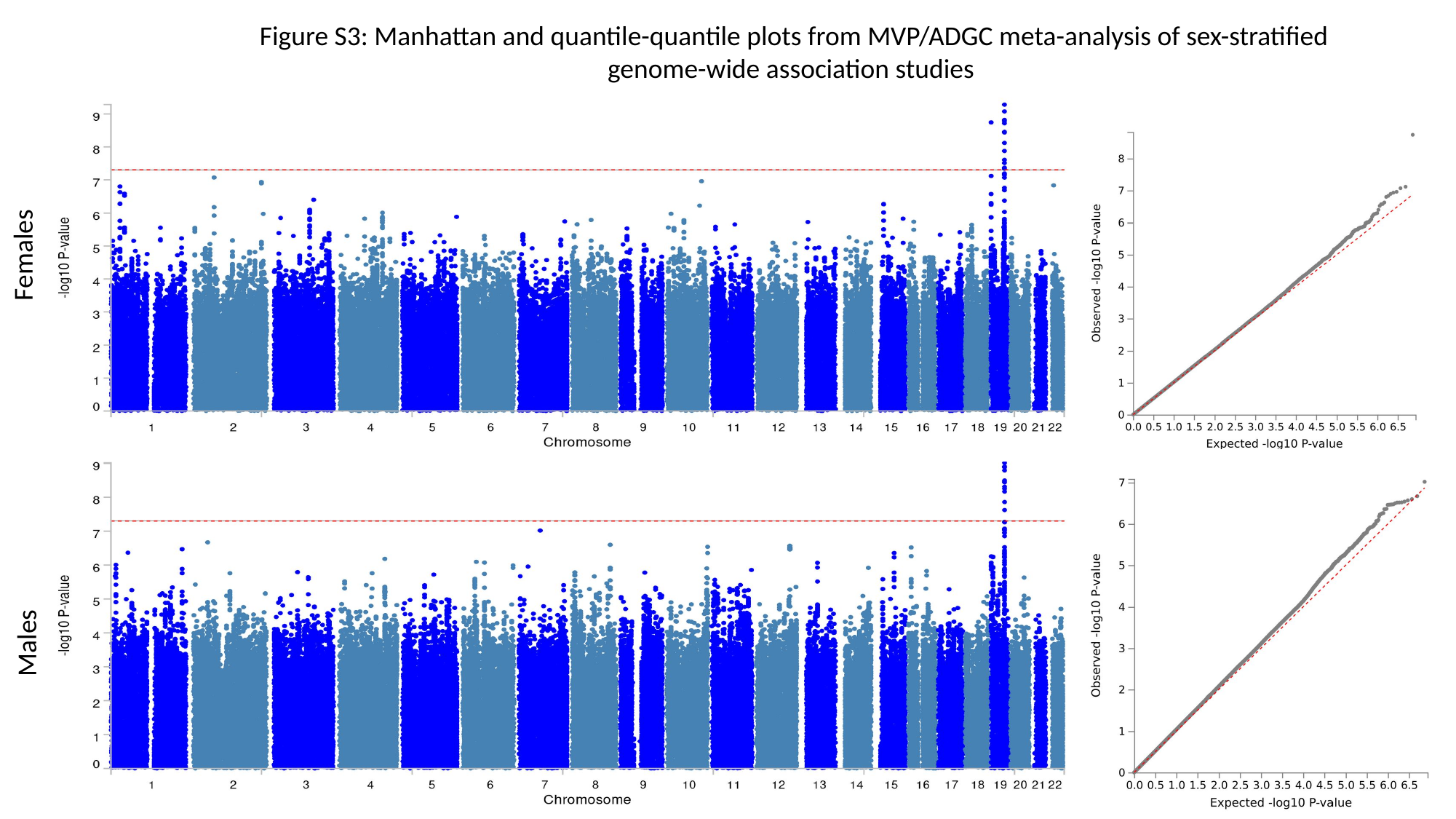

Figure S3: Manhattan and quantile-quantile plots from MVP/ADGC meta-analysis of sex-stratified genome-wide association studies
Females
Males

## Slide 4
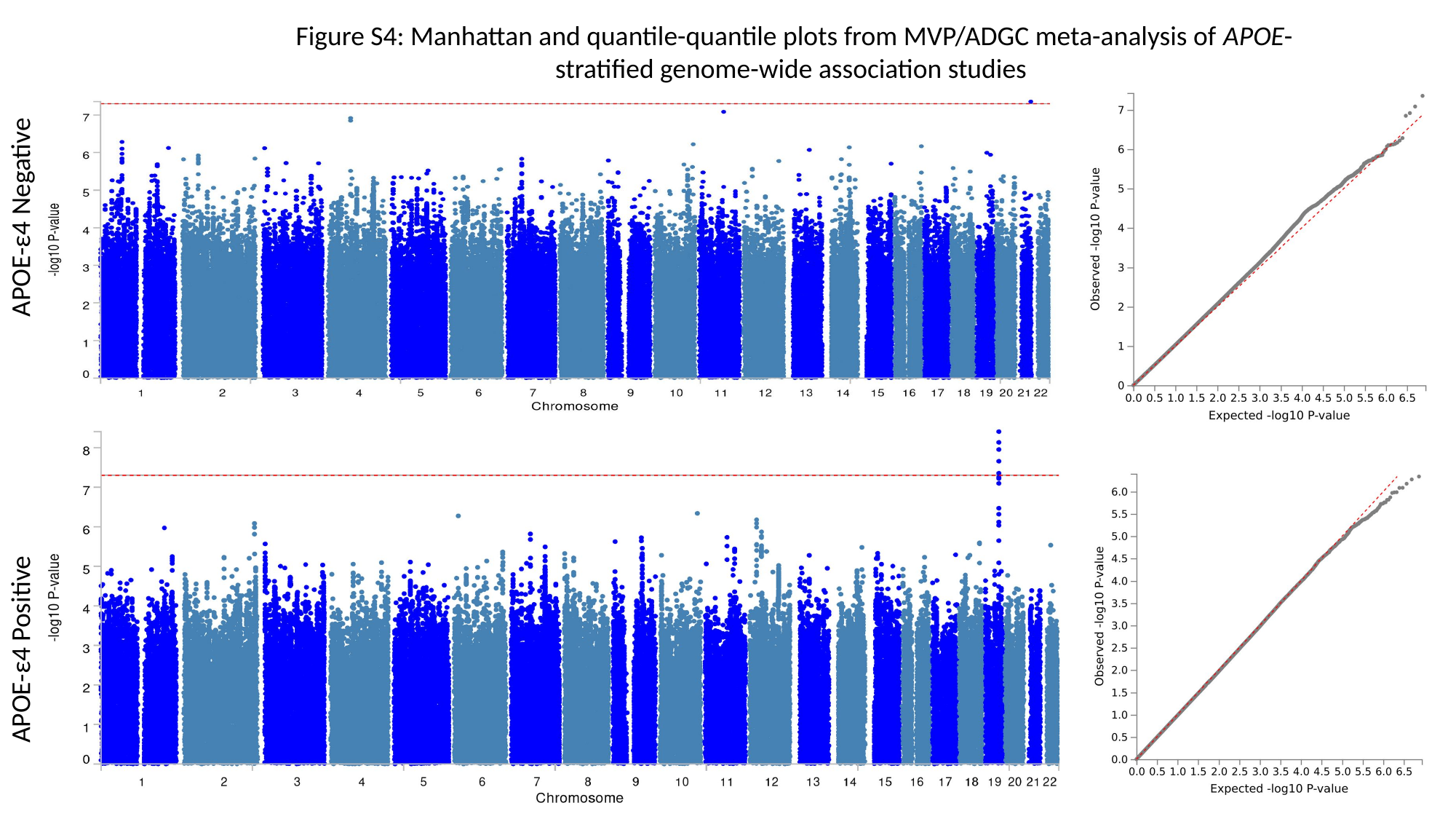

Figure S4: Manhattan and quantile-quantile plots from MVP/ADGC meta-analysis of APOE-stratified genome-wide association studies
APOE-ε4 Negative
APOE-ε4 Positive
